# Cell turnover in the repopulated rat liver: distinct lineages for hepatocytes and the biliary epithelium

**DOI:** 10.1007/s00441-014-1800-5

**Published:** 2014-04-01

**Authors:** Fabio Marongiu, Maria Paola Serra, Marcella Sini, Michela Marongiu, Antonella Contini, Ezio Laconi

**Affiliations:** Department of Biomedical Sciences, Unit of Experimental Medicine, University of Cagliari School of Medicine, Via Porcell 4, 3rd Floor, Cagliari, 09124 Italy

**Keywords:** Liver repopulation, Progenitor cells, Cell turnover, Hepatocyte transplantation, Streaming liver, Rat

## Abstract

The dynamics of cell renewal in the normal adult liver remains an unresolved issue. We investigate the possible contribution of a common biliary precursor cell pool to hepatocyte turnover in the chimeric long-term repopulated rat liver. The retrorsine (RS)-based model of massive liver repopulation was used. Animals not expressing the CD26 marker (CD26^-^) were injected with RS, followed by transplantation of 2 million syngeneic hepatocytes isolated from a normal CD26-expressing donor. Extensive (80-90 %) replacement of resident parenchymal cells was observed at 1 year post-transplantation and persisted at 2 years, as expected. A panel of specific markers, including cytokeratin 7, OV6, EpCAM, claudin 7 and α-fetoprotein, was employed to locate the *in situ* putative progenitor and/or biliary epithelial cells in the stably repopulated liver. No overlap was observed between any of these markers and the CD26 tag identifying transplanted cells. Exposure to RS was not inhibitory to the putative progenitor and/or biliary epithelial cells, nor did we observe any evidence of cell fusion between these cells and the transplanted cell population. Given the long-term (>2 years) stability of the donor cell phenotype in this model of liver repopulation, the present findings suggest that hepatocyte turnover in the repopulated liver is fuelled by a cell lineage distinct from that of the biliary epithelium and relies largely on the differentiated parenchymal cell population. These results support the solid biological foundation of liver repopulation strategies based on the transplantation of isolated hepatocytes.

## Introduction

The liver exhibits slow parenchymal cell turnover. Estimates of the average lifespan of hepatocytes under non-perturbed conditions are in the range of 1 year, although the exact quantification of this parameter has been elusive so far, particularly in the human liver (MacDonald 1961; Ponder 1996; Imai et al. 2001). Paradoxically, the liver is also endowed with a proliferative capacity, as exemplified both clinically and experimentally by the intense regenerative response that follows partial surgical hepatectomy. Indeed, the ability to compensate for cell loss is an important functional feature of hepatocytes, given their potential physiological exposure to diet-related toxic and metabolic insults (Alison 1986; Michalopoulos 2010).

In spite of its relevance to liver physiology, the source of hepatocyte turnover remains an unsettled issue. The large majority of differentiated hepatocytes are nevertheless known to be capable of undergoing cell division following massive tissue loss, implying that a specialized tissue niche devoted to hepatocyte renewal is not needed (Duncan et al. 2009). On the other hand, several lines of evidence indicate that hepatocytes can be generated from progenitor cell populations when the replicative capacity of the resident differentiated compartment is impaired or exhausted, thus supporting the contention that a precursor cell type for hepatocytes does indeed exist in the adult liver, including normal human liver (Schmelzer et al. 2007; Semeraro et al. 2012; Español-Suñer et al. 2012). However, the biological mechanisms involved in the continuous, albeit slow, daily replenishment of lost hepatocytes under normal steady-state conditions remains to be firmly established.

Almost 30 years ago, Zajicek et al. (1985) proposed the streaming liver hypothesis, which postulates that hepatocytes are normally generated in the periportal space of the liver acinus (zone 1) and move slowly along the sinusoids to end up and die in the pericentral area (zone 3). The entire journey has been estimated to last about 1 year, based on data derived from the analysis of normal rat liver.

Subsequent studies have not been supportive of the above proposition. For example, Bralet et al. (1994) observed no evidence of migratory behavior in retrovirally tagged hepatocytes after 1 year of follow up in rats and similar results have been reported in mice (Kennedy et al. 1995). Based on these findings, the hypothesis of hepatocyte streaming has become less attractive, although recent evidence provided by Furuyama et al. (2011) has reawakened interest in this concept. The normal turnover of mouse hepatocytes has been suggested to be sustained by intrahepatic biliary epithelial cells and to follow a pattern consistent with the continuous streaming of parenchymal cells from the periportal area to the central hepatic vein, with an overall transit time of about 1 year. Data compatible with a migratory behavior of hepatocytes along the sinusoidal direction of blood flow had also been obtained in earlier studies conducted on human liver (Fellous et al. 2009). The results of Furuyama et al. (2011) have however been recently questioned (Malato et al. 2011).

Given these conflicting findings and considering the relevance of this issue to the field of regenerative medicine (Alison et al. 2012), we explore the origin of hepatocyte turnover in a model of massive liver repopulation. In rats exposed to retrorsine (RS) and then injected with normal syngeneic hepatocytes, near-total replacement of the resident parenchyma by donor-derived cells is observed (Laconi et al. 2001). Notably, liver repopulation in this system is stable, persisting for at least 2 years after cell transplantation (Laconi et al. 2006). The latter observation is important, because it implies that any rate of hepatocyte turnover in these animals must be sustained by cells of donor origin, irrespective of their specific phenotype. Furthermore, if hepatocyte streaming does occur in the repopulated liver, it should be fuelled by a donor-derived precursor cell pool. The present studies were designed to test the above possibilities. The results indicate the hepatocyte turnover is sustained by a cell lineage distinct from that of the biliary epithelium and relies largely on the transplanted differentiated parenchymal cell population.

## Materials and methods

### Animals and treatments

The dipeptidyl-peptidase-deficient (DPPIV^-^) Fischer 344 rat model (Thompson et al. 1991) was used for transplantation experiments. A colony of DPPIV^-^ animals was available in the animal room at the University of Cagliari, whereas donor DPPIV^+^ F344 rats were purchased from Charles River, Italy. All animals were maintained under alternating 12 h light/dark daily cycles and were fed *ad libitum* with Rodent Chow Diet.

Experiments were approved by the University of Cagliari Ethical Committee for Animal Experimentation and were in accordance with NIH Guidelines for the care and use of animals (NIH publication 86–23, revised 1985). Six-week-old DPPIV^-^ rats (with a body weight of 70-90 g) were treated with two i.p. injections of either 30 mg/kg RS (Sigma) or saline, 2 weeks apart. Four weeks after the last injection of RS, eight animals were transplanted, via the portal circulation, with 2 million normal hepatocytes isolated from a DPPIV^+^ donor. Groups of four rats each were killed 1 or 2 years after cell transplantation. Another group of four rats was given RS only and was killed 1 year later. Livers were excised and samples from each lobe were either snap-frozen (for cryostat sections) or fixed in 10 % buffered formalin and embedded in paraffin.

Histochemical determination of DPPIV enzyme activity and quantitation of percent repopulation were performed as described (Laconi et al. 2001) on three samples taken from each lobe.

In a parallel study, groups of 4 control and 4 RS-treated animals did not receive hepatocyte transplantation and were subjected to 2/3 partial hepatectomy (PH) 4 weeks after the initial treatment. All rats were killed 48 h post-PH; each animal was injected with 5′-bromo-deoxyuridine (BrdU, 50 mg/kg, i.p.), starting at 20 h post-surgery and every 8 h until being killed. The uptake of BrdU was expressed as the percent of labeled cells. At least 20 biliary ducts were scored in each animal.

### Hepatocyte isolation and transplantation

Hepatocytes for transplantation were isolated from a 6-week-old DPPIV+syngeneic F344 rat according to a standard two-step collagenase perfusion technique (Berry and Friend 1969; Seglen 1976). Cell viability was >85 % as assessed by trypan blue dye exclusion. Hepatocytes were suspended in phosphate-buffered saline (PBS; 1 × 10^7^/ml) and delivered through a branch of the mesenteric veins.

### Immunohistochemistry and immunofluorescence

Immunohistochemical staining for DPPIV/CD26 was performed on formalin-fixed sections following antigen retrieval in boiling citrate buffer (0.1 M, pH 6) for 15 min. Sections were then incubated with the primary antibody (BD, San Jose, Calif., USA) overnight at 4 °C and then with alkaline-phosphatase-conjugated secondary antibody. Detection of a specific signal was accomplished by using the avidin/biotin/alkaline phosphatase system (Vectastain ABC kit; Vector Labs, Burlingame, Calif., USA). Immunohistochemical staining for Ki67 was carried out on 5-μm-thick frozen sections fixed in 0.1 % acetic acid/ethanol, washed in PBS and blocked with goat serum. Primary antibody (Abcam, Cambridge, Mass., USA) was applied overnight at 4 °C and then samples were processed as described for paraffin sections.

Immunofluorescent staining was performed on 5-μm-thick frozen sections that had been fixed for 10 min in cold acetone. Primary antibodies were: cytokeratin 7 (CK7), EpCAM, α-fetoprotein (αFP), Claudin 7, Ki67 (Abcam), DPPIV/CD26, BrdU (Dako, Denmark) and OV6 (kindly provided by Dr. Dabeva). Slides were blocked with goat serum, incubated with primary antibodies for 1 h at room temperature, followed by incubation for 45 min with fluorescent-conjugated secondary antibodies (Dylight 488, Abcam; Alexa Fluor 555, Life Technologies, Grand Island, N.Y., USA). Slides were finally covered with Vectashield mounting medium with 4,6-diamidino-2-phenylindole (DAPI; 1 μl/ml; Vector Labs, UK) to visualize cell nuclei. Images were acquired with an IX71 fluorescence microscope with a charge-coupled device camera (Olympus, Tokyo, Japan).

One to three samples were taken from each liver lobe. All sections were thoroughly screened for the expression of the relevant markers (see above).

### Enzyme histochemistry

Histochemical determination of DPPIV enzyme activity was performed as described by Laconi et al. (2001). Briefly, cryostat sections were fixed in 0.1 % acetic acid/ethanol, air-dried and incubated for 15 min at room temperature with the substrate reagent, namely 2.5 mg Gly-Pro-4-methoxy-β-naphthylamide dissolved in 150 μl dimethylformamide and then mixed with 5 ml of a solution of Fast blue BB salt (1 mg/ml in PBS). All reagents were purchased from Sigma.

## Results

### Transplanted cells do not replace resident bile ductular epithelium

**Fig. 1:**
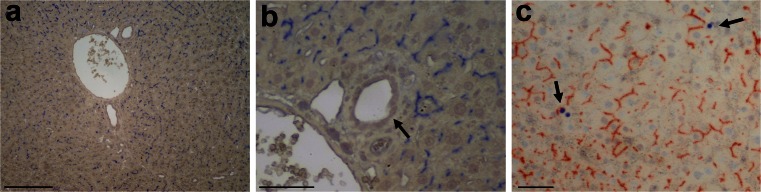
Stability of donor-derived phenotype in retrorsine (RS)-treated repopulated liver. **a**, **b** Immunohistochemical staining for DPPIV/CD26 antigen in the liver of a RS-treated DPPIV^-^ rat transplanted with hepatocytes isolated from a DPPIV^+^ syngenic donor. The sample was taken 2 years after cell transplantation. Note the persistent replacement of the DPPIV^-^ host liver by DPPIV-expressing hepatocytes (*dark blue*), with the typical chicken-wire pattern associated with this enzyme protein. Bile ductular epithelium (**b** *arrow*) does not express DPPIV and maintains the original host phenotype. **c** Section of liver sample from the same group of animals and stained for histochemical detection of DPPIV (*orange-rust*) and expression of Ki67 antigen (immunohistochemistry, *dark blue*). Note the presence of scattered rare nuclei positive for the cell cycle marker (*arrows*). *Bars* 200 μm (**a**), 50 μm (**b**, **c**)

As previously mentioned, the main version of the streaming liver hypothesis posits that hepatocytes originate near the periportal space from a precursor pool that is shared with the epithelium of the biliary tree. To test this possibility, the origin of such a putative common precursor cell compartment was analyzed in the repopulated liver. We first confirmed and extended our earlier observation concerning the stability of the donor hepatocyte phenotype in the RS-exposed and repopulated liver (Laconi et al. 2006). As shown in Fig. 1, 80-95 % repopulation of the RS-treated DPPIV^-^ rat liver by DPPIV^+^ donor-derived hepatocytes was observed as late as 24 months post-transplantation (Fig. 1a, b). Similar results were seen in animals killed after 1 year (data not presented). The rate of hepatocyte turnover in the repopulated liver was evaluated through the immunohistochemical detection of Ki67 antigen. Rare labeled hepatocytes (about 1/5000) were seen throughout the liver parenchyma, with no apparent zonal distribution (Fig. 1c). Interestingly, biliary epithelial cells in periportal areas did not express the DPPIV enzyme (Fig. 1a, b) indicating that they retained the host phenotype and were not replaced by donor-derived cells.

**Fig. 2:**
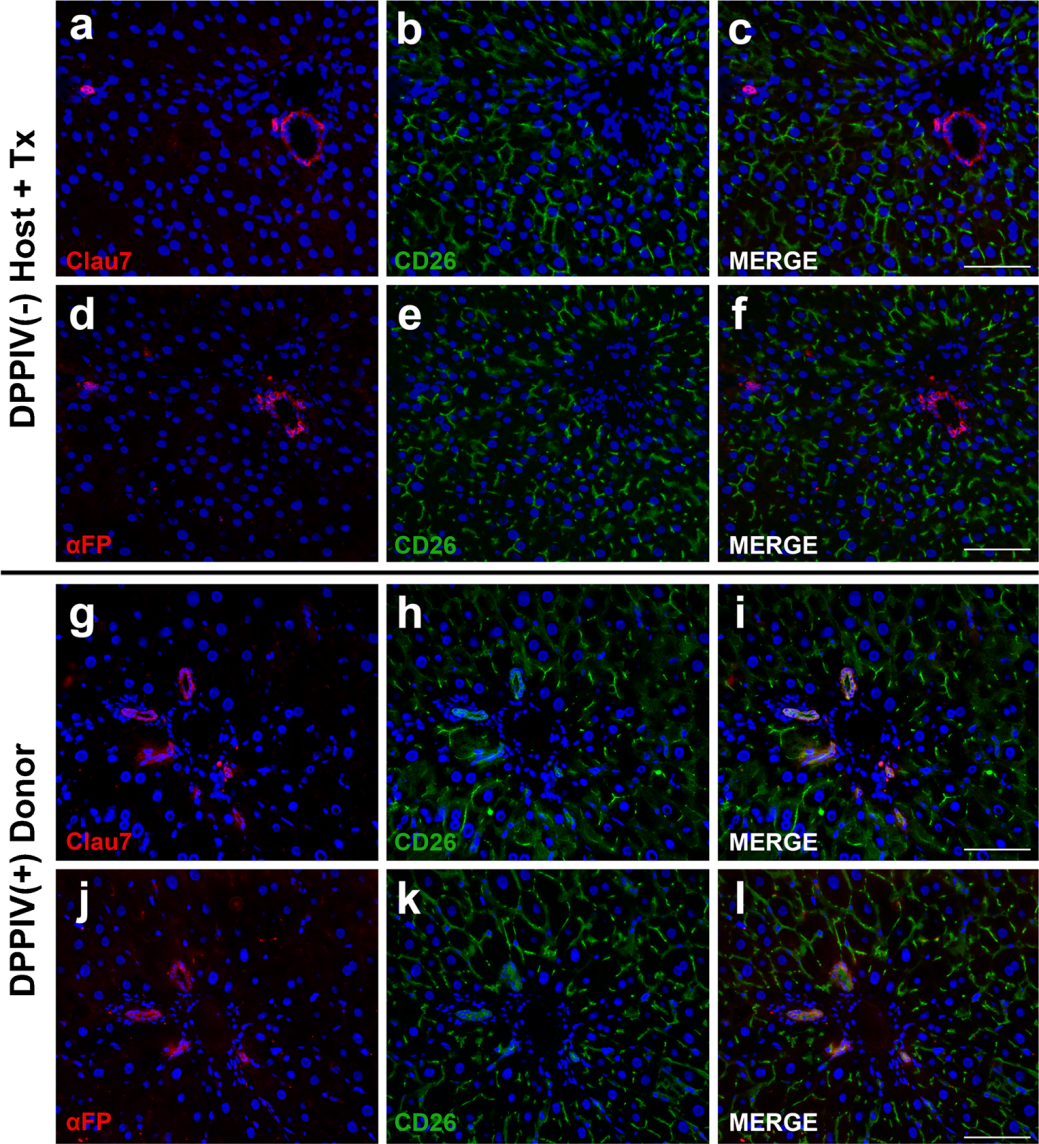
Claudin 7 (*Clau7*)- or α-fetoprotein (*αFP*)-positive cells do not express the CD26 donor cell marker (*blue* nuclear staining with DAPI). Dual immunohistochemical staining for either claudin 7 (**a–c**) or αFP (**d–f**) and DPPIV/CD26 (*CD26*) in the liver of RS-treated DPPIV^-^ rat transplanted with hepatocytes isolated from a DPPIV^+^ syngenic donor (*+Tx* plus transplant). Samples were taken 1 year post-transplantation. No overlap was observed between DPPIV/CD26 expression and staining for claudin 7 or αFP. Note the extensive repopulation of the host liver (*green*) around claudin 7- or αFP-expressing cells. For comparison, **g–l** show co-expression of DPPIV/CD26 in claudin 7- or αFP-positive cells from normal untreated DPPIV^+^ rats. *Bars* 100 μm

**Fig. 3:**
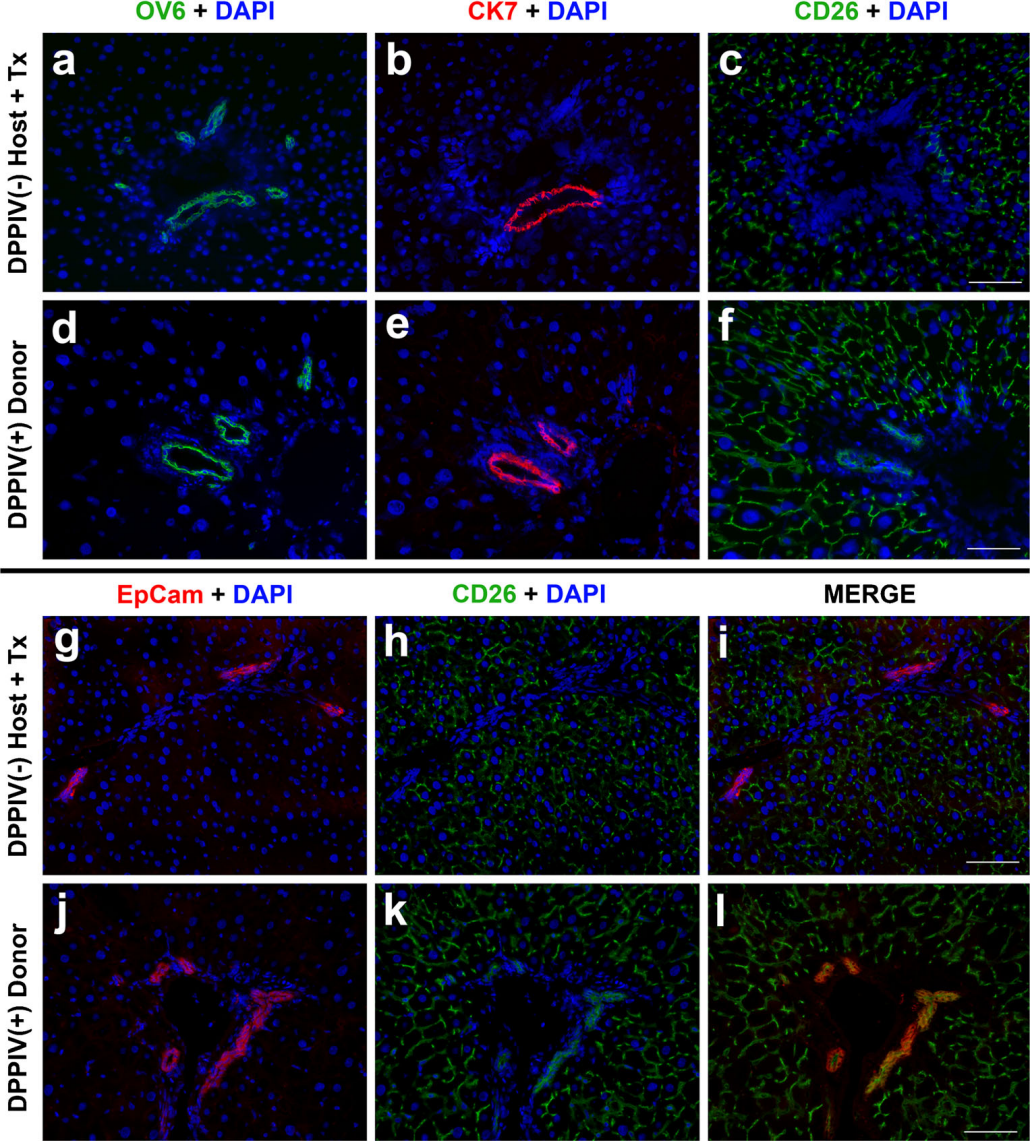
OV6-, CK7-, or EpCAM-positive cells do not express the CD26 donor cell marker. **a–c** Immunofluorescence staining for OV6, CK7 and DPPIV/CD26, respectively, on serial sections obtained from RS-treated DPPIV^-^ rat liver transplanted with hepatocytes isolated from a DPPIV^+^ syngenic donor. Samples were taken 1 year post-transplantation. Note that OV6- and/or CK7-positive cells do not express the donor DPPIV/CD26 marker. **d–f** Immunofluorescence staining for OV6, CK7 and DPPIV/CD26, respectively, on serial sections obtained from untreated DPPIV^+^ rat liver. Note that both OV6- and CK7-positive cells also express DPPIV/CD26 antigen. **g–i** Dual immunohistochemical staining for EpCAM and DPPIV/CD26 in the liver of RS-treated DPPIV^-^ rat transplanted with hepatocytes isolated from a DPPIV^+^ syngenic donor. Samples were taken 1 year post-transplantation. No overlap was observed between DPPIV/CD26 expression and staining for EpCAM. For comparison, **j–l** show co-expression of DPPIV/CD26 in EpCAM-positive cells from normal untreated DPPIV^+^ rats. *Bars* 100 μm

The latter finding was then explored in greater detail by using a panel of immunohistochemical markers for the *in situ* detection of biliary epithelial cells and/or putative hepatocyte progenitor cells in the repopulated liver. Markers included CK7, OV6, EpCAM, claudin 7 and α-FP (Figs. 2, 3). Positive staining for claudin 7 was observed in periportal bile ducts and in rare isolated cells within the lobule of repopulated livers in animals killed 1 year after hepatocyte transplantation (Fig. 2a). Extensive liver repopulation was documented by the diffuse expression of CD26/DPPIV enzyme, a marker of donor-derived cells (Fig. 2b). No overlap was seen between CD26- and claudin-positive cells (Fig. 2c) indicating that the latter were of recipient origin. This conclusion was supported by the co-expression of CD26 and claudin 7 in the bile ductular epithelium of DPPIV-positive Fischer rats (Fig. 2g–i).

Similar findings were observed following immunofluorescent staining for α-FP (Fig. 2d–f), OV6 (Fig. 3a), CK7 (Fig. 3b) and EpCAM (Fig. 3g). All markers were expressed by the periportal biliary epithelium and by isolated cells or small cell clusters dispersed in the liver lobule, although CK7 appeared to be specific for large bile ducts. No overlap between any of these markers and CD26 was observed. On the other hand, the large majority of surrounding hepatocytes expressed CD26, confirming their origin from donor-derived cells (Fig. 2b, c, e, f, Fig. 3c, h, i). Importantly, claudin 7, α-FP, OV6, CK7 and EpCAM were each co-expressed with CD26 in the bile ductular epitheliums of DPPIV-positive animals, as expected (Fig. 2g–l, Fig. 3d–f, j–l).

### Exposure to RS does not inhibit proliferation of bile ductular epithelium

**Fig. 4:**
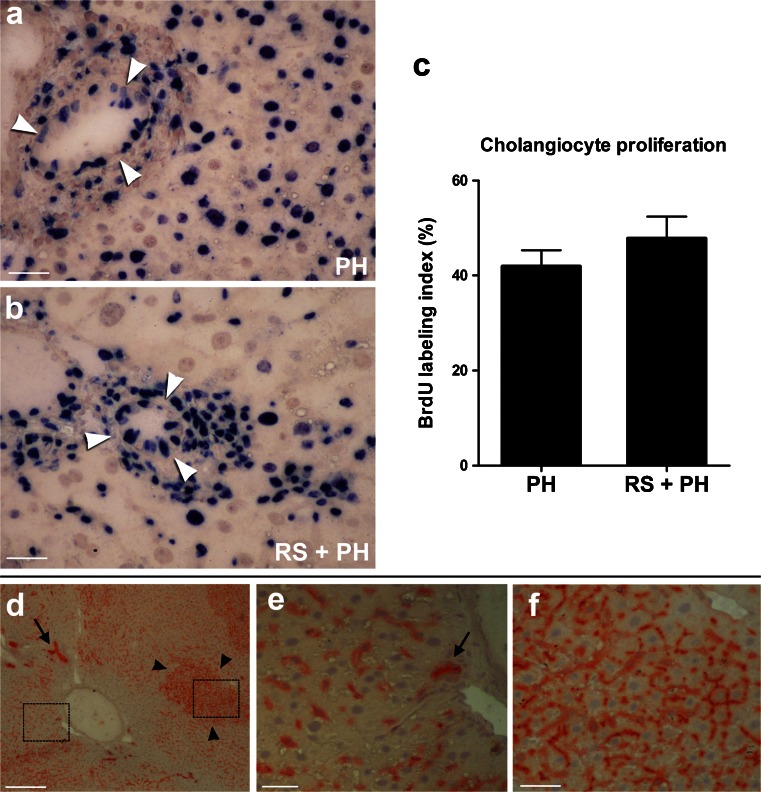
No evidence of cell fusion during RS-induced liver repopulation. **a–c** RS does not inhibit proliferation of bile ductular epithelial cells (*arrowheads* dividing cells). Immunohistochemical staining for 5′-bromo-deoxyuridine (*BrdU*) uptake in liver sections obtained from control (**a**) or RS-treated rats (**b**) at 2 days after partial hepatectomy (*PH*). Quantitation of BrdU-labeling index is shown in **c**. Original magnification 200×. **d–f** No evidence of cell fusion during RS-induced liver repopulation. Histochemical staining for DPPIV enzyme activity (*orange-rust*) in the liver of a RS-treated DPPIV^+^ rat transplanted with hepatocytes isolated from a DPPIV^-^ syngenic donor. The sample was taken 1 year after cell transplantation. The DPPIV^+^ host liver was extensively repopulated by DPPIV^-^ hepatocytes (**d**). However, non-parenchymal cells, including bile ductular epithelium (*arrows*), maintained the original phenotype of the host liver and expressed DPPIV (*left box* in **d**, enlarged in **e**). Isolated areas of DPPIV-expressing hepatocytes were also observed, possibly representing endogenous regenerative nodules (*arrowheads*, *right box* in **d**, enlarged in **f**). *Bars* 50 μm (**a**, **b**, **e**, **f**), 200 μm (**d**)

The findings described above rule out the involvement of a common biliary precursor pool in sustaining hepatocyte turnover in the RS-treated and repopulated liver. One possible explanation for this result would be that RS inhibits the proliferation of bile ductular epithelium. We therefore tested this possibility in rats subjected to 2/3 partial hepatectomy following exposure to RS. Immunohistochemical staining for the uptake of BrdU revealed comparable levels of staining in the biliary epithelium from RS-treated and control animals (Fig. 4a–c) suggesting that no specific inhibitory effect was exerted by RS on the bile ductular cell cycle.

### Hepatocyte fusion with putative progenitor cells is not a major factor in RS-induced liver repopulation

Cell fusion is considered to occur physiologically in the liver (Wang et al. 2003; Quintana-Bustamante et al. 2006; Faggioli et al. 2008). However, the possible contribution of this process to liver repopulation remains a debated issue. Given the stability of the donor hepatocyte phenotype in the RS-treated and repopulated liver, one can still postulate that putative endogenous progenitor cells (DPPIV-) might be involved in hepatocyte turnover through fusion with differentiated transplanted hepatocytes (DPPIV+), resulting in the prevalence of the latter phenotype. To address this possibility directly, we reversed the phenotype of the donor and the recipient animals by transplanting DPPIV- hepatocytes into RS-treated DPP-IV+ hosts. Under these conditions, one would expect a prevalent DPPIV+ phenotype among the hepatocytes of the repopulated liver, if indeed cell fusion had a prominent role during the process. However, the observed results did not support this possibility. We found instead that the RS-treated DPPIV+ host liver was extensively repopulated by donor-derived cells expressing their original DPPIV- phenotype (Fig. 4d). This indicated that fusion of transplanted hepatocytes with putative endogenous progenitor cells was not a prominent phenomenon, if indeed it occurred at all. Scattered areas of host-derived (DPPV+) hepatocytes were also present, possibly representing endogenous regenerative nodules, which are known to occur in RS-treated liver (McLean 1970; (Fig. 4d, left box, enlarged in e). The results presented also indicated that hepatocyte transplantation in RS-exposed rats replaced only the parenchymal component of the liver, whereas other cell types, including bile ductular epithelium, were still of recipient origin and expressed the DPPIV enzyme marker (Fig. 4d, right box, enlarged in f).

## Discussion

Taken together, our present findings support the conclusion that long-term hepatocyte turnover in the repopulated rat liver is largely fuelled by differentiated transplanted cells of donor origin and that the cell lineage is distinct from that of the biliary epithelium. This conclusion is based on the following: (1) the donor-cell phenotype of the repopulated liver remains stable over at least 2 years, as previously shown (Laconi et al. 2006) and as confirmed by the present studies; (2) the transplanted cells replace only the parenchymal component (hepatocytes) of the host liver, whereas other cell types, including biliary epithelium, remain of recipient origin; (3) no cross-reactivity can indeed be documented between the donor cell marker (DPPIV/CD26) and a panel of markers specific for biliary epithelium and/or putative progenitor cells in the repopulated rat liver; (4) no evidence of cell fusion between transplanted hepatocytes and putative endogenous progenitor cells has been observed.

The rate of hepatocyte turnover under normal steady-state conditions has been difficult to assess: estimates in rodent liver vary between 200 and 450 days but they have been as high as 3000 days (MacDonald 1961; Ponder 1996; Imai et al. 2001). The general agreement is that hepatocytes are long-lived cells, despite their enormous potential to replicate in response to appropriate stimuli (MacDonald 1961; Ponder 1996; Imai et al. 2001; Alison 1986; Michalopoulos 2010). Our present data on the extent of Ki67 labeling in hepatocytes in the stably repopulated rat liver are in agreement with this conclusion, confirming the low proliferative rate of this tissue in the adult/aged animal.

In these studies, we also ruled out the possibility that treatment with RS impairs the replicative potential of a putative common precursor cell pool, thereby preventing its contribution to hepatocyte turnover. Unlike genetically based models of liver repopulation, such the urokinase-plasminogen-activator (uPA) transgenic mouse (Rhim et al. 1994) and the fumaryl-acetoacetate-hydroxylasae (FAH) defective mouse (Overturf et al. 1996), RS-treated animals bear no driver alterations in the germ line. Thus, cell types in the liver, other than hepatocytes, need not be affected by RS in their proliferative capacity. Not surprisingly, we observed that cells expressing the biliary epithelium exhibit a similar response to PH in both RS-treated and control rats, indicating no direct inhibitory effect of the alkaloid on the proliferation of these cells. In this context, we should also take into consideration that exposure to pyrrolizidine alkaloids, including RS, has long been associated with the emergence of liver regenerative nodules (McLean 1970; Schoental and Magee 1959). The cell of origin of these nodules is still debated, with at least three different hypothesis having been proposed (Best and Coleman 2007; Pichard et al. 2009; Chen et al. 2013); however, their presence *per se* indicates that RS does not suppress the activation of a progenitor cell compartment that is indeed able slowly to replace RS-induced megalocytes in a process that we have referred to as endogenous repopulation (Laconi et al. 2008). As is apparent from the present studies, transplanted hepatocytes expressing a fully differentiated phenotype are able to outrun the growth of the endogenous progenitor cell pool following RS-induced injury, resulting in the long-term repopulation of the host liver with a donor-derived phenotype.

Finally, we addressed the possible involvement of cell fusion between donor and recipient cells during hepatocyte turnover of the repopulated liver. Several reports have documented the occurrence of both homotypic (Faggioli et al. 2008) and heterotypic (Wang et al. 2003; Quintana-Bustamante et al. 2006) fusion events in the liver. Thus, the possibility that putative endogenous progenitor cells could fuse with transplanted hepatocytes, generating hybrid heterocaryons of mixed phenotype, had to be tested. To this end, we reversed the donor and recipient phenotypes in the DPPIV model of hepatocyte transplantation, i.e., we transplanted DPPIV-deficient hepatocytes into RS-treated DPPIV^+^ host livers. We observed that the recipient parenchyma was efficiently repopulated by transplanted hepatocytes and that the latter maintained the donor-derived DPPIV^-^ phenotype. We also confirmed that non-parenchymal cells, including bile ductular epithelium, were not replaced by the transplanted cell population, indicating that the RS-induced process of liver repopulation is largely tissue-specific (Michalopoulos et al. 2005).

In summary, our present results indicate the presence of two distinct cell lineages sustaining hepatocyte and biliary cell turnover in the repopulated liver. In light of these findings, the long-term stability (>2 years) of the donor-derived phenotype in this model of liver repopulation is consistent with a continuous, albeit extremely slow, generation of new hepatocytes from the transplanted differentiated cell population. Remarkably, this occurs in spite of the presence of an endogenous RS-induced pool of progenitor cells (Schoental and Magee 1959; Best and Coleman 2007; Pichard et al. 2009; Chen et al. 2013; Laconi et al. 2008).

To our knowledge this is the first study exploring the origin of hepatocyte turnover in a long-term, stably repopulated liver. With respect to the relevance of this issue to the field of regenerative medicine, these results support the solid biological foundation of liver repopulation strategies based on the transplantation of isolated hepatocytes.
